# Protective effects of hesperidin in cyclophosphamide-induced parotid toxicity in rats

**DOI:** 10.1038/s41598-022-26881-w

**Published:** 2023-01-04

**Authors:** Ola A. Abdelwahab Mostafa, Fatma Ibrahim, Eman Borai

**Affiliations:** 1 Department of Anatomy and Embryology, Faculty of Medicine, Zagazig University, Zagazig, 44519 Egypt; 2 Department of Forensic Medicine and Clinical Toxicology, Faculty of Medicine, Zagazig University, Zagazig, Egypt

**Keywords:** Anatomy, Biomarkers, Endocrinology, Medical research, Pathogenesis

## Abstract

Cyclophosphamide (CYP) is an alkylating agent that is used on a wide range as a treatment of malignancies and autoimmune diseases. Previous studies have shown the promising role of hesperidin (HSP) as an antioxidant agent against various models of toxic agents. The protective effect of the HSP against CYP-induced parotid damage was evaluated in this study. Forty rats (180–200 g) were divided into four equal groups: Group I (received normal saline), Group II (HSP-treated at a dose of 100 mg/kg/day for 7 consecutive days), Group III (CYP-treated at a dose of 200 mg/kg single intraperitoneal injection on the 7th day of the experiment), Group IV (CYP + HSP); HSP-treated at a dose of 100 mg/kg/day for 7 consecutive days and CYP (200 mg/kg) single intraperitoneal injection on the 7th day of the experiment. Afterwards, the oxidative stress and inflammatory markers, the histopathological and immunohistochemical alterations of the parotid tissues in the studied groups were evaluated. CYP intoxication induced a significant parotid tissue injury represented by the elevation in the values of malondialdehyde (MDA), tumor necrosis factor-α (TNF-α) and interleukin-1β (IL-1β) and decrease in the catalase activity and glutathione peroxidase (GPx). Histologically, extensive histopathological alterations e.g., widely spaced serous acini with irregular shapes and congested blood vessels as well as downregulated ki-67 and alpha-smooth muscle actin (α-SMA) immunoexpression were induced by CYP. HSP administration markedly improved the biochemical and the histopathological studies. We can conclude that HSP elicited protective effects against the CYP-induced parotid toxicity.

## Introduction

Cyclophosphamide (CYP) is a widely-used chemotherapeutic drug. It is also used for treatment of autoimmune diseases e.g. systemic lupus^1^. Though the increased oxidative stress and inflammation caused by it restricted its several clinical uses^2^. Nausea, vomiting, alopecia, pancytopenia, infections, hemorrhagic cystitis, and malignancies are the most common known CYP- induced adverse effects^3,4^.

According to previous research studies, CYP is reported to cause multi-organ toxicities for example; the immune system^5^, male reproductive system^6^, female reproductive system^7^, the liver^8^, and the kidney^9^.

Several changes occur to the oral cavity throughout the intensive cancer chemotherapy courses. Decreased saliva production resulting in xerostomia^10^ and oral mucositis^11^ are reported adverse effects of CYP.

Xerostomia affects chewing, swallowing and speech functions, which can induce nutritional insufficiencies and impaired social activity. It is responsible for an increased frequency of oral candidiasis and dental caries, all of these effects can impair the daily life of the patient, which is requiring additional care of the oral cavity^12^. Consequently, the parotid glands have a significant importance as they produce about 60–65% of the total saliva^13^.

The effect of CYP on the functions of the salivary gland seems to be extended to months or sometimes years even after cessation of treatment. However, most studies assessed salivary gland function only during the treatment course with no longer follow-up after that, which is a point of limitation^14^.

The effect of CYP on the salivary glands generally and the parotid glands specifically has been listed in many research articles. This effect is existent in nearly all cases receiving CYP with variable degrees depending on the dose and the duration of the treatment course, the patient’s age, and general condition as well^14,15^.

CYP undergoes metabolism by liver cytochromes P450, specifically  cytochrome 3A4 and  cytochrome 2B6^16^, generating two active metabolites: phosphoramide mustard and acrolein, which is responsible for its detrimental toxic effects^17^.

Several mechanisms have been suggested to explain CYP-induced toxicity. CYP causes cross-linking of DNA bases, thereby inhibiting DNA replication and inducing apoptosis^18^. Acrolein can enter the cells causing activation of the intra cellular reactive oxygen species (ROS) and nitric oxide production, forming peroxynitrite which eventually damages the intracellular lipids, proteins and DNA^19^.

Hesperidin (HSP) is a natural flavonoid that is isolated from citrus plants such as grapefruit, lemon and orange^20^. It is supposed to have antioxidant, anti-carcinogenic, and anti-inflammatory properties^21,22^. It has been used in protection as well as treatment of many toxicity models e.g. cisplatin, methotrexate, carbon tetrachloride and carbon monoxide^20,23–25^.

Hesperidin effectively increases the activities of superoxide dismutase, catalase and glutathione peroxidase resulting in powerful elimination of ROS along with decreasing free radicals production and lipid peroxidation^26^.

To our knowledge, this is the first research to reveal the valuable role of HSP against CYP-induced parotid injury. Consequently, this work was implented to investigate the possible defensive role of HSP against CYP-provoked parotid injury through an evaluation of biomarkers of oxidative stress, inflammatory process, along with the histopathological and immunohistochemical examination of parotid tissue.

## Materials and methods

### Materials

Cyclophosphamide (CYP) was purchased from Sigma–Aldrich Chemicals Co., St. Louis, USA. Hesperidin (HSP) as a pale yellow powder, was purchased from Sigma˗Egypt, (Cairo, Egypt). CYP and HSP were dissolved in normal saline to be administered to rats.

### Experimental protocol

The study included 40 adult male albino rats, each weighing 180–200 g. Two weeks before starting the experiment, all rats were exposed to passive preliminaries to be adapted to their new surroundings, to determine their physical well-being and to dismiss any diseased rats. The Institutional Animal Care and Use Committee (IACUC), Zagazig University approved this study (Approval number: ZU-IACUC/3/F/3/2022). All methods were performed in accordance with the relevant guidelines and regulations.

This research included four equal experimental groups, with ten rats in each one. Group I (Control): received normal saline for seven days.

Group II (HSP): treated with HSP (100 mg/kg body weight) daily orally for seven consecutive days. Selection of the dose was based on prior study of Berköz et al.^27^.

Group III (CYP): treated with CYP (200 mg/kg body weight) single intraperitoneal injection on the 7th day of the experiment. Selection of the dose was based on prior study of Taslimi et al.^28^.

Group IV (CYP + HSP): treated with HSP (100 mg/kg body weight) daily orally for seven consecutive days and CYP (200 mg/kg body weight) single intraperitoneal injection on the 7th day of the experiment.

### Blood and parotid preparation

At the end of the treatment protocol, blood samples were collected from the retro-orbital venous plexus in clean plain test tubes followed by centrifugation at 3000 rpm for 15 min to separate the sera, which were stored at − 20 °C until assessment of tumor necrosis factor-α (TNF-α) and interleukin-1β (IL-1β). Then, all animals were sacrificed; parotid glands were dissected and weighed. Then the tissues were distributed into two parts. The first one was homogenized and stored at − 80 °C for measuring of catalase activity, the levels of malondialdehyde (MDA) and glutathione peroxidase (GPx). The second part was prepared for histopathology and immunohistochemistry.

### Biochemical markers

Malondialdehyde (Bio diagnostic, CAT. No. MD 25-29), catalase activity (Biodiagnostic, CAT. No. CA 25-17) and glutathione peroxidase (Biodiagnostic, CAT. No. GP 25-24) were assayed using the methods of Ohkawa et al., Fossati et al. and Paglia and Valentine^29–31^ respectively.

Tumor necrosis factor-α and interleukin-1β were assessed by ELISA kits, according to the manufacturer’s recommended methods (CUSABIO, CAT. No. CSB-E11987r and CSB-E08055r).

### Histological study

#### Histological  examination

Rat parotid tissue samples were automatically processed and embedded in paraffin blocks after being instantly fixed in formalin, dried in graded ethanol, cleaned in xylene, and embedded. A microtome was used to chop up samples of parotid tissues into 4–5 m slides. Hematoxylin and eosin (H&E) and Mallory's trichrome were used to stain the sections to evaluate general tissue histology and collagen fibers distribution^32,33^. 10 fields from 5 sections from each rat in each group were coded permitting blind examination and evaluation using light microscope and ImageJ software.

Mallory’s trichrome sections were assessed at 400 magnifications for the mean area percentages of blue-stained collagen fiber (CF) and red-counter stained smooth muscle (SM) within the musculosa^34^.

#### Immunohistochemical and morphometrical analysis

Paraffin-embedded 5 μm slices of rat parotid were treated with rabbit monoclonal anti-Ki-67 and the primary antibody anti-E-cadherin (Zymed, San Francisco, CA, USA) overnight at 4 °C (Abcam plc, Cambridge, UK). After applying the secondary antibody, a chromogen was then added (3,30-diaminobenzidine). Finally, Mayer hematoxylin stain was used as a counterstain on tissue sections. Each rat in each group had 10 fields from 5 sections that were coded to allow for blind analysis and evaluation.

Ki-67 immunostained sections were assessed at 400 magnifications for calculation of the proliferation index (PI), by calculating the number of positive cells (brown cells) %^35^.

Avidin–biotin complex was added to the sections. They were incubated at 4 °C overnight, deparaffinized and rehydrated by sequences of alcohol of different grades. Then the sections were boiled in sodium citrate and immersed in 3% H_2_O_2_ to stop the activity of the endogenous peroxidase. The slices were then treated with primary antibodies for 1 h at room temperature after being incubated with 10% normal goat serum to prevent non-specific antibody binding, and then they were incubated with primary anti-a-SMA (purchased from BioGenex). Biotinylated anti-rabbit antibody (Vector Laboratories, Burlingame, CA, USA) was added to the sections for 30 min at room temperature. The antigen–antibody complex was detected using streptavidin–biotin–peroxidase. Finally, 3,3-diaminobenzidine substrate was added to the sections then they were counterstained with Mayer’s hematoxylin^36^. The morphometrical analysis was performed using Leica Q 500 MC program at the Anatomy and Embryology Department, Faculty of Medicine, Zagazig University.

### Statistical analysis

The measured parameters in the studied groups were expressed as mean ± standard deviation (mean ± SD), and were compared with each other using IBM SPSS Statistics, version 24 (IBM; Armonk, New York, USA). One-way analysis of variance (ANOVA), followed by post-Hoc Tukey were performed to detect statistical differences among groups. When the *P* value (probability of chance) was < 0.05, the differences were considered significant, whereas, *P* ≥ 0.05 showed a non-significant difference. All statistical comparisons were two-tailed.

### Ethical approval

All experimental methods were authorised by Zagazig University's Institutional Animal Care and Use Committee (ZU-IACUC), with approval number (ZU-IACUC/3/F/3/2022), and all were carried out in accordance with ARRIVE recommendations.

## Results

### Mortality rates

No deaths were recorded in any of the studied groups.

### Body weight changes and relative parotid weight

As shown in Table 1, when comparing the treated (CYP) group to both control and (HSP) groups, the mean values of body weight and the parotid weight revealed a non-significant decrease. There was no significant difference between both (control and HSP groups) and (CYP + HSP) group.

**Table 1:** Effects of hesperidin on cyclophosphamide regarding initial body weight, final body weight, parotid weight in the studied groups.

Parameter	Groups			
	Control	HSP	CYP	HSP + CYP
Initial body weight (g)	186.80 ± 6.53	188.7 ± 2.67	186.50 ± 5.70	187.3 ± 4.86
Final body weight (g)	210.80 ± 6.41	211.50 ± 3.31	206.00 ± 2.94	211.70 ± 5.62
Parotid weight (mg)	98.10 ± 1.80	98.50 ± 3.41	95.30 ± 2.87	98.20 ± 3.23

All values are presented as mean ± SD, n = 10. *P* value ≥ 0.05 is non-significant.

*HSP* Hesperidin, *CYP* Cyclophosphamide.

### Biochemical markers

This study showed that there were no statistically significant differences in mean values of serum MDA, catalase activity, GPx, TNF‐α and IL-1β in the HSP group in comparison to the control group (P > 0.05).

The results shown in Table 2 revealed that CYP significantly increased MDA that is indicative of increased oxidative stress in CYP-induced injury of the parotid gland. Alternatively, the catalase activity and GPx were significantly decreased as compared to the resultant values in the control. The HSP administration had protective effects against these changes as it significantly reduced the levels of MDA and significantly elevated the catalase activity and GPx in the group exposed to CYP.

**Table 2:** Effects of hesperidin on cyclophosphamide regarding the oxidative stress parameters in the studied groups.

Parameter	Groups			
	Control	HSP	CYP	HSP + CYP
Tissue MDA (nmol/g)	91.14 ± 2.63	88.34 ± 3.29	135.50 ± 3.52^a,b^	98.10 ± 0.97^a,b,c^
Tissue Catalase (U/g)	19.44 ± 1.28	18.65 ± 1.16	6.91 ± 0.56^a,b^	13.29 ± 0.30^a,b,c^
Tissue GPx (ng/mg)	22.08 ± 2.02	25.06 ± 4.24	13.82 ± 3.14^a,b^	18.22 ± 1.65^a,b,c^

All values are presented as mean ± SD, n = 10. *P* value < 0.05 is significant. Post hoc Tukey’s multiple comparisons test following one-way ANOVA expressed as letters: ^a^significant versus control, ^b^significant versus HSP, ^c^significant versus CYP.

*MDA* Malondialdehyde, *GPx* Glutathione peroxidase, *HSP* Hesperidin, *CYP* Cyclophosphamide.

As shown in Table 3, TNF-α and IL-1β levels were significantly elevated in rats treated with CYP as compared to the control and HSP groups. The HSP administration prior to CYP resulted in significantly decreased the inflammatory parameters (TNF-α and IL-1β) when compared to the CYP group.

**Table 3:** Effects of hesperidin on cyclophosphamide regarding the inflammatory parameters in the studied groups.

Parameter	Groups			
	Control	HSP	CYP	HSP + CYP
Serum TNF-α (pg/ml)	11.01 ± 2.37	13.23 ± 1.19	24.56 ± 3.60^a,b^	18.74 ± 0.56^a,b,c^
Serum IL-1β (pg/ml)	22.67 ± 1.53	23.53 ± 2.56	51.86 ± 2.01^a,b^	33.31 ± 1.30^a,b,c^

All values are presented as mean ± SD, n = 10. *P* value < 0.05 is significant. Post hoc Tukey’s multiple comparisons test following one-way ANOVA expressed as letters: ^a^significant versus control, ^b^significant versus HSP, ^c^significant versus CYP.

*TNF‑α* Tumor necrosis factor‑alpha, *IL-1β* Interleukin-1β, *HSP* Hesperidin, *CYP* Cyclophosphamide.

### Histological  results

#### Hematoxylin and eosin stain

**Figure 1:**
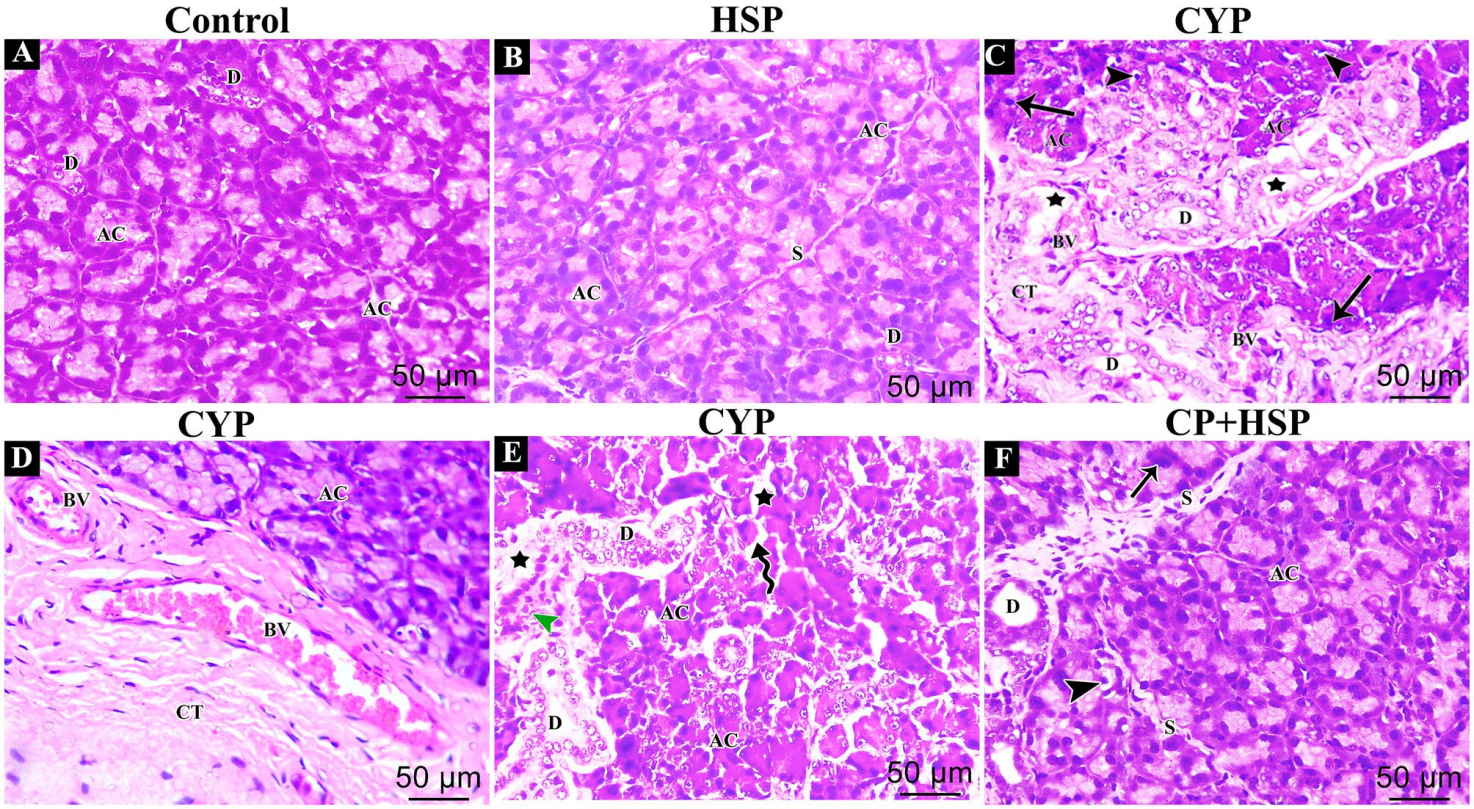
(**A**–**F**) photomicrographs of H&E stained sections of the parotid gland of rats of different studied groups. (**A**,**B**) control and HSP groups displaying the normal parenchyma as tightly clustered exclusively serous acini (AC) and ducts (D) separated by thin septa (S). (**C**–**E**) CYP group showing disorganized acini (AC) with disappearance of their lumens. Most acinar cells lining these acini have pyknotic or darkly stained nuclei (thin arrow) while other cells are vacuolated (arrow head). These acini are widely  separated (star) with excess connective tissue fibers (CT). Dilated congested blood vessels (BV) and extensively dilated irregular Ducts (D) with absent secretion are observed. Notice also periductal cellular infiltration (Green arrow head). (**F**) CYP + HSP group showing restoration of the normal structure with pure serous acini packed closely together (AC). Some vacuolated acinar cells (arrow head)  and pyknotic cells with darkly stained nuclei (thin arrow) are still occasionally seen with slightly dilated ducts (D). Septa (S) are slightly thin. (H&E × 400).

Upon examination of H&E stained sections of both control and HSP groups, a normal parotid gland histological structure was found. Purely serous acini together with some hardly noticeable ducts between these acini made up the parotid parenchyma. These serous acini and ducts were closely packed together and separated by thin septa (Fig. 1A,B).

The parotid tissues were disorganized in the CYP group. The majorities of the serous acini were widely spaced apart and had irregular shapes. Also expanded congested blood vessels together with overgrowth of connective tissue fibers were observed between these widely spaced distorted acini. Variable-sized vacuoles displaced the nuclei in their periphery, and small pyknotic nuclei were also seen while the ducts in some acini displayed significant dilatation and secretion stagnation. Additionally, periductal cellular infiltration was seen (Fig. 1C–E).

In the CYP + HSP group, the parotid tissues exhibited some improvement. Purely serous acini appeared closely packed in the parotid parenchyma. There were still some vacuolations. The ducts displayed some dilatation (Fig. 1F).

#### Mallory staining

**Figure 2:**
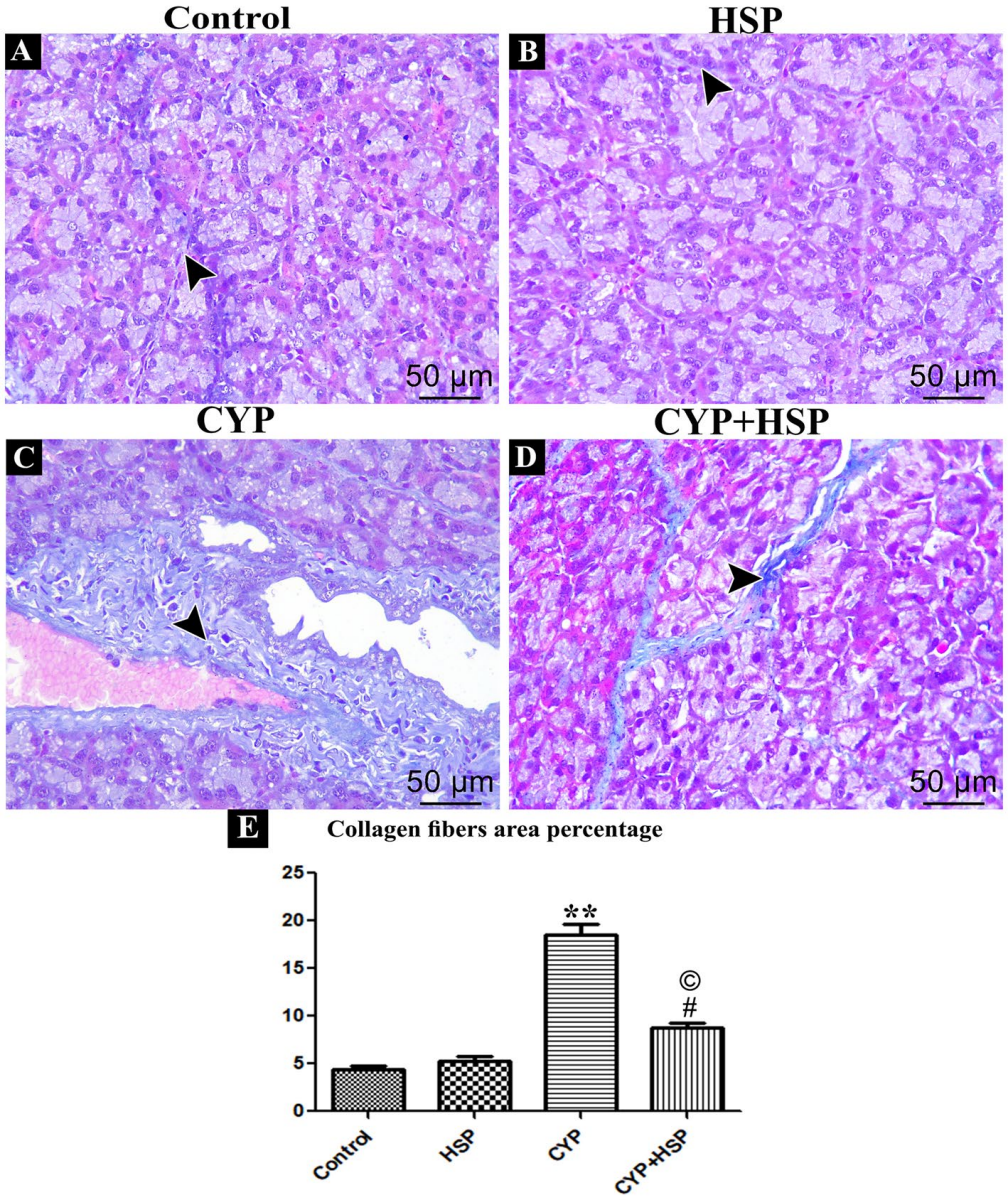
(**A**–**D**) photomicrographs of Mallory stained sections of the parotid gland of rats of different studied groups showing collagen fibers (arrow head). (**A**,**B**) control and HSP groups showing few collagen fibers in-between acini. (**C**) CYP group showing excess amounts of collagen fibers in between acini and around the dilated congested blood vessels. (**D**) CYP + HSP group showing slightly moderate amounts of collagen fibers in between acini. (**E**) A bar chart showing morphometrical image analysis for collagen fibers area percentage. (**) high significant difference in CYP group vs both control and HSP groups. (#) significant difference in CYP + HSP group vs both control and HSP groups. (©) significant difference in CYP + HSP group vs CYP group. (Mallory Trichromex 400).

There was very little collagen fibers between the acini in the parotid glands of rats in both control and HSP groups in Mallory stained parotid gland sections (Fig. 2A,B). While there were excessive amounts of collagen fibers around the dilated blood vessels and between the deformed acini in the CYP group (Fig. 2C). Moderate amounts of collagen fibers were detected in the CYP + HSP group (Fig. 2D).

Moreover, morphometric image analysis for collagen fibers area percentage in CYP group revealed that there was highly significant increase vs. both control and HSP groups, also after HSP administration; there was a significant difference in CYP + HSP group as compared to both (control and HSP) groups and CYP group (Fig. 2E).

#### Immunohistochemical staining for Ki-67

**Figure 3:**
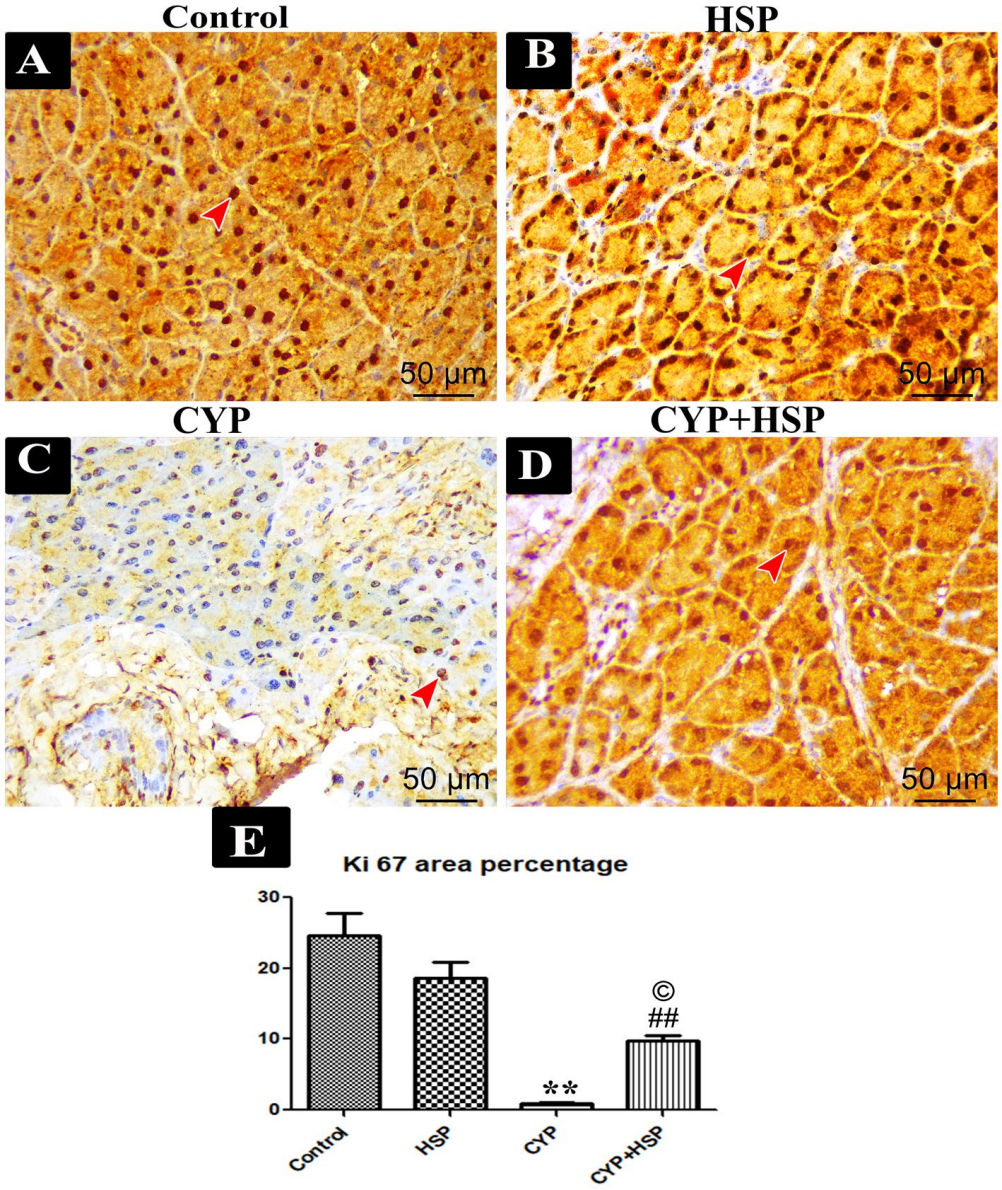
(**A**–**D**) Photomicrographs of immunoreactivity for Ki67 of the parotid gland sections of rats of different studied groups. (**A**,**B**) Control and HSP groups showing strong positive stained nuclei taking brown color (red arrow head) while in (**C**) CYP group showing very little amount of positive stained nuclei. (**D**) CYP + HSP group showing slightly moderate amounts of positive stained nuclei taking brown color (red arrow head). (**E**) A bar chart showing morphometrical image analysis for Ki67 area percentage. (**) high significant difference in CYP group vs both control and HSP groups. (##) high significant difference in CYP + HSP group vs both control and HSP groups. (©) significant difference in CYP + HSP group vs CYP group. (Immunohistochemistry Ki67 × 400).

The acinar cells of both control and HSP groups had a strong positive nuclear immunoreactivity for Ki-67 (Fig. 3A,B). While the acinar cells of the CYP group had a very little positive nuclear immunoreactivity for Ki-67 (Fig. 3C). Moderate amounts of Ki-67 positively reacted nuclei were detected in the CYP + HSP group (Fig. 3D).

Morphometric image analysis for Ki-67 in the CYP group revealed that there was a highly significant decrease vs. both control and HSP groups. There was a high significant difference in the CYP + HSP group as compared to both control and HSP groups and also a significant difference when compared to CYP group (Fig. 3E).

#### Immunohistochemical staining for alpha smooth muscle actin (α-SMA)

**Figure 4:**
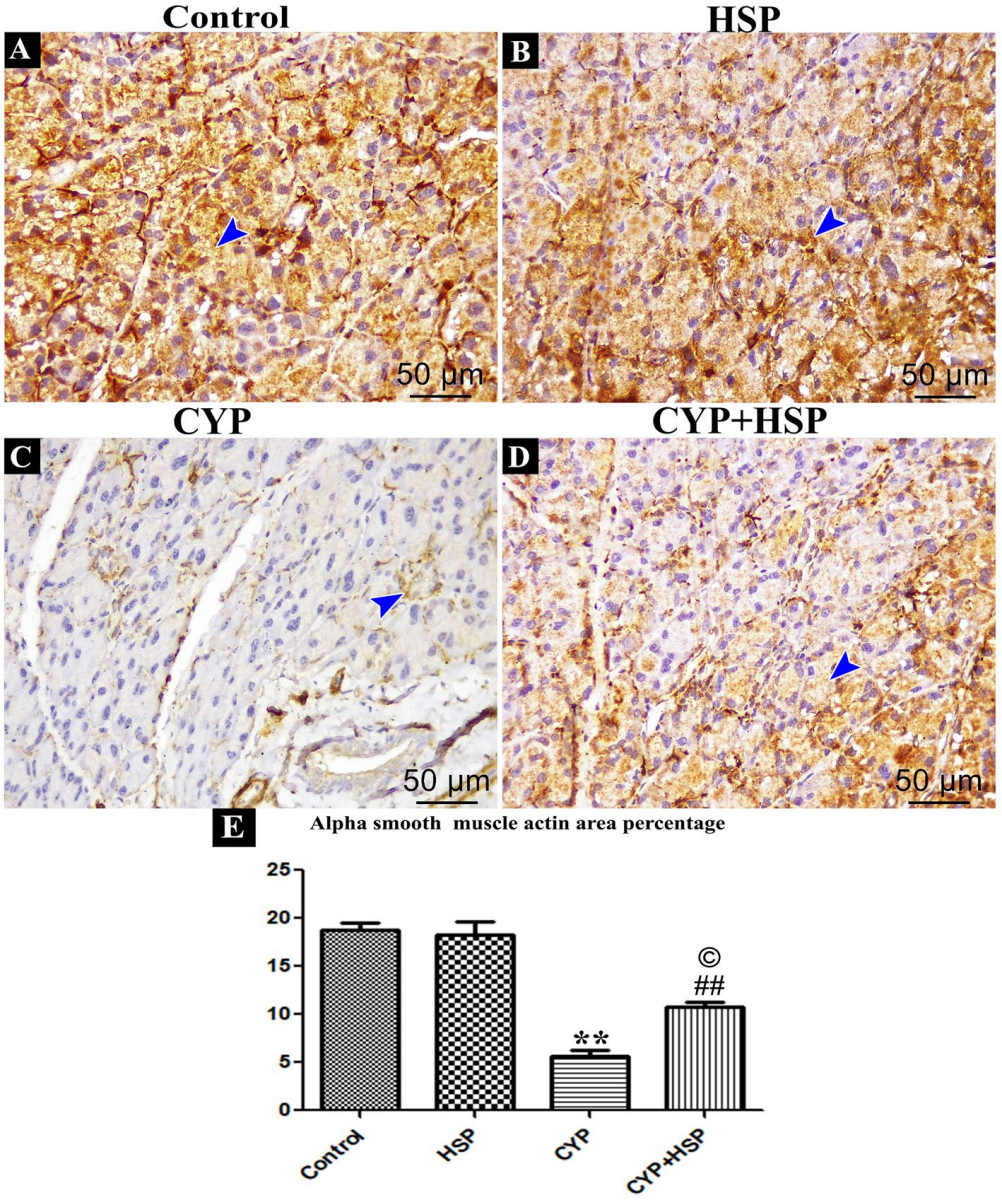
(**A**–**D**) Photomicrographs of immunoreactivity for  alpha smooth muscle actin. Positive stained cytoplasm is taking brown color (blue arrow head). (**A**,**B**) control and HSP groups. (**C**) CYP group. (**D**) CYP + HSP group. (**E**) A bar chart showing morphometrical image analysis for α-SMA area percentage. (**) high significant difference in CYP group vs both control and HSP groups. (##) high significant difference in CYP + HSP group vs both control and HSP groups. (©) significant difference in CYP + HSP group vs CYP group. (Immunohistochemistry for alpha smooth muscle sctin (α-SMA) × 400).

Both control and HSP groups exhibited noticeably higher levels of positive cytoplasmic immunohistochemical expression of acinar cells for α-SMA (Fig. 4A,B). In contrast, cytoplasm of the CYP group acinar cell displayed pronounced weak immunohistochemical expression (Fig. 4C). The CYP + HSP group displayed moderate positive cytoplasmic immunohistochemical expression for α-SMA (Fig. 4D).

Additionally, morphometric image analysis for α-SMA in the CYP group revealed that there was a high significant decrease vs. both control and HSP groups. There was a high significant difference in CYP + HSP group as compared to both control and HSP groups. Moreover, a high significant difference when compared to CYP group (Fig. 4E).

## Discussion

Many previous in vivo and in vitro studies reported the toxic effects of CYP. It has been proposed that CYP causes detrimental effects in many organs, including the salivary glands^37,38^.

The present results showed that CYP provoked inflammatory and oxidative stress effects in the parotid glands as well as histopathological and immunohistochemical changes. However, HSP had an ameliorative effect on all studied parameters.

In this study, CYP induced elevation in the MDA levels as well as decreased catalase activity and GPx levels when compared to the controls, which coincides with the findings of previous studies^39,40^.

Acrolein is the active metabolite of CYP, produced by the liver microsomal enzymes^17^. It is responsible for its toxic effects as it causes disruption of mitochondrial oxidative phosphorylation and production of ROS^41^.

Malondialdehyde level is primarily used for assessment of the status of lipid peroxidation and oxidative membrane damage^42^.

The findings of the present study regarding GPx and catalase correlate with Kim et al.^43^, as they are crucial antioxidant enzymes and the principal scavengers of H_2_O_2_, which keeping them as a cellular defense mechanism against ROS^44^.

In the current study, CYP induced a significant increase in the levels of TNF-α which is matched with the findings of Khodeer et al.^45^, who reported the same results. In addition, Ohtani et al.^46^ reported that 4-hydroxycyclophosphamide (4-HC), which is a metabolite of CYP augments the TNF-α mediated DNA fragmentation.

Tumor necrosis factor-α induces tissue‐specific inflammation through the involvement of ROS generation and triggering of various transcriptional mediated pathways^47^.

As presented in the current study’s results, CYP induced a significant increase in the IL-1β levels, which is parallel to the findings of previous studies^48–50^.

Continuous CYP-provoked ROS production can trigger stress signaling and pro-inflammatory pathways activation with subsequent release of pro-inflammatory cytokines, including IL-1β^51^.

Histopathological findings in this study coincide with the published studies of Yahyazadeh and Alnuaimi et al.^38,52^, who stated the toxic effect of CYP on the salivary gland cells, the stroma, the ducts, and the acini. Chemotherapeutic drugs like CYP could affect the function of acinar and ductal cells, by disrupting the cell division^53^.

Acrolein has the ability to induce the breakdown of the intraluminal membrane, facilitating its penetration of the underlying epithelium along with initiating an inflammatory process, causing edema, neutrophil infiltration, hemorrhage and devastating tissue damage^54,55^.

In the parotid, fibroblasts synthesize collagen and secrete it in a soluble form to be deposited extracellularly, either decreased collagen breakdown or an increased biosynthesis may affect the occurring fibrosis. CYP-caused tissue fibrosis could be attributed to increased hydroxyproline, which is a keystone in the fibrosis^56,57^, which explains the excess amounts of collagen fibers seen in the Mallory stained sections in the CYP group.

Ki-67 is a nuclear protein that is associated with cellular proliferation. It was reported that chemotherapeutic agents caused decrease in its expression^58^.

Other studies^59–61^ reported the anti-proliferative effects of CYP on different tissues either healthy or malignant possibly due to the arrest of the cell cycle as CYP has been shown to suppress mitosis and cell replication by binding to DNA^62^. It is valuable to mention that healthy cells has higher affinity to uptake CYP more than the malignant cells, which make the former more vulnerable to injury^63^.

In the current study, CYP caused a decreased α-SMA labeling in the parotid glands. α-SMA is a protein existing in the microfilaments of myoepithelial cell cytoskeleton of the parotid glands^64^. The decrease in α-SMA could be accompanied with complications in saliva production and excretion, resulting in xerostomia^65^.

Many antioxidants and nutritional supplementations have been used in experimental models to counteract CYP-induced toxicity with most of them recording positive results^66–68^.

In the current study, HSP ameliorated the toxic effects of CYP on the parotid gland. Co-administration of HSP resulted in decreased MDA levels besides increased catalase activity and GPx levels when compared to the controls. Also, the HSP was effective in decreasing the concentrations of TNF-α and IL-1β. In addition, HSP caused improvement of the histopathological and immunohistochemical alterations triggered by CYP.

Hesperidin has been reported to have favorable effects against various oxidative stress states caused by toxins^69–71^. HSP can act as a powerful scavenger of ROS as well as enhancing the antioxidant defenses of the cell, which make it an effective antioxidant^21^.

Hesperidin has been reported to have direct anti-inflammatory properties through modifying the Nf-κB pathway, which is affected by CYP^72,73^.

The improved biochemical parameters were reflected in the histopathological and the immunohistochemical study as the normal structure of the parotid gland tissue was preserved along with increased reaction to Ki67 and α-SMA in HSP co-treated group, which is supported by previous studies that suggested the ameliorative effect of HSP against CYP-induced damage of various organs^74–77^.

## Conclusion

Cyclophosphamide provoked damage in the parotid tissue as evidenced by elevated values of MDA, TNF-α and IL-1β and decreased catalase activity and GPx, along with extensive histopathological changes in the parotid tissue as well as down regulated ki-67 immunoexpression and α-SMA immunoexpression. The present study revealed for the first time that the HSP administration could be helpful in reducing CYP-induced parotid damage.

## Data Availability

This published article [and its additional information files] contains all data produced or analyzed during this investigation. The corresponding author will provide the datasets used and/or analyzed during the current work upon reasonable request.
